# Variable-Barrier Quantum Coulomb Blockade Effect in Nanoscale Transistors

**DOI:** 10.3390/nano12244437

**Published:** 2022-12-13

**Authors:** Pooja Yadav, Soumya Chakraborty, Daniel Moraru, Arup Samanta

**Affiliations:** 1 Quantum/Nano-Science and Technology Lab, Department of Physics, Indian Institute of Technology Roorkee, Roorkee 247667, India; 2 Research Institute of Electronics, Shizuoka University, 3-5-1 Johoku, Naka-ku, Hamamatsu 432-8011, Japan; 3 Centre of Nanotechnology, Indian Institute of Technology Roorkee, Roorkee 247667, India

**Keywords:** quantum dot, donor atom transistor, single electron transistor, Coulomb blockade, variable tunnel barrier

## Abstract

Current–voltage characteristics of a quantum dot in double-barrier configuration, as formed in the nanoscale channel of silicon transistors, were analyzed both experimentally and theoretically. Single electron transistors (SET) made in a SOI-FET configuration using silicon quantum dot as well as phosphorus donor quantum dots were experimentally investigated. These devices exhibited a quantum Coulomb blockade phenomenon along with a detectable effect of variable tunnel barriers. To replicate the experimental results, we developed a generalized formalism for the tunnel-barrier dependent quantum Coulomb blockade by modifying the rate-equation approach. We qualitatively replicate the experimental results with numerical calculation using this formalism for two and three energy levels participated in the tunneling transport. The new formalism supports the features of most of the small-scaled SET devices.

## 1. Introduction

Advancement in nano-fabrication techniques for the development of silicon (Si) nanoscale devices has provided a valuable platform for the realization and investigation of sophisticated devices that can transfer electrons with higher efficiency and accuracy than the typical metal-oxide-semiconductor field-effect transistors (MOSFETs) [1,2,3,4,5,6]. Some of these devices, namely the single-electron transistors (SETs), exploit the physics of Coulomb blockade (CB) as the basic operational principle. These exotic devices can have target functionalities towards logic circuits [7], single-electron memories [8], single-charge sensing [9], charge- and spin-based quantum computing [10,11], single electron pump [12], single photon detector [13], highly sensitive biosensors [14], etc. A double-barrier quantum dot (QD) geometry formed within such SETs can periodically suppress single electron transfer due to the subsequent charging energy. This phenomenon is generally known as the Coulomb blockade [15,16,17,18]. Initially, SETs were studied in metallic QDs, where the discreteness of the energy levels within the QD can be ignored [19,20,21,22,23]. However, in nanoscale semiconductor-based SETs, where the energy separation between successive discrete energy levels within the QD is comparable to or higher than the thermal energy, the scenario is different from the classical Coulomb blockade and is known as quantum Coulomb blockade (QCB). In this QCB regime, single electron passes through discrete energy levels of the QD. Such phenomenon is generally observed for nano-scaled SET devices fabricated in two-dimensional electron gas (2DEG) systems, semiconductor QDs and dopants as QDs [24,25,26,27,28,29].

The initial theoretical framework for QCB had been put forward by C.W.J. Beenakkar [30], which is valid mainly for the linear-response regime. The general procedure to analyze the nonlinear regime was pioneered and carried out previously using rate-equation approach for the SETs with QDs in infinite-barrier configurations [29,31,32]. However, practical SETs have finite potential barriers, and the gate also typically tunes the heights of the tunnel barriers due to limitations of the nano-fabrication techniques. Several experimental reports have already shown the effects of gate-dependent potential barriers [9,33,34,35,36,37,38,39]. However, the theoretical formalism for describing such results has still not been explored sufficiently and its numerical model still requires further development. 

In this article, we present example of variable barrier in experimental results for devices with QDs formed in the Si nanoscale channels along with an extended theoretical treatment of the rate-equation approach to apply this to the quantum Coulomb blockade regime for SETs with variable tunnel barriers. Starting from the Hamiltonian for a QD coupled to source, drain and gate electrodes, we review the tunneling rates via each tunnel junctions, discussing in detail the assumptions and our notations under which this approach has significant accuracy. We also extend the rate-equation approach for the observation of quantum Coulomb blockade phenomenon in an N-level QD system having constant and variable tunnel barrier and finally, the numerical models for such systems are outlined comparatively.

## 2. Materials and Methods

### 2.1. Experimental Devices

We present experimental result for two devices, namely *Device-A* and *Device-B*. All devices are fabricated in silicon-on-insulator (SOI) substrates, using standard complementary metal-oxide-semiconductor (CMOS) fabrication processes, as described in other works [36,37,39]. The SOI substrates used here had a top Si-layer with a thickness *t*_SOI_ ≈ 10 nm and a gate oxide with a thickness *t*_ox_ ≈ 10 nm, while the source, drain and gate electrodes are formed by aluminum contact.

**Figure 1:**
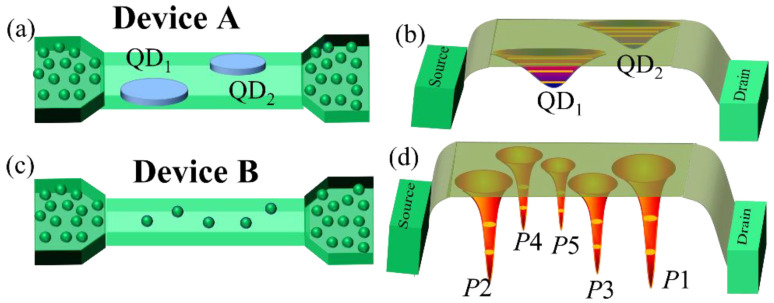
(**a**,**b**) Schematic device structure and schematic potential configuration of *Device-A* (with a nominally undoped nanoscale channel). (**c**,**d**) Schematic device structure and schematic potential configuration of *Device-B* (with uniformly doped channel).

Figure 1a shows the channel region of *Device-A*, which represents a nanoscale SOI-FET having no intentional doping in the channel region. However, some degree of surface roughness may induce broad QDs in the channel, with potential wells schematically shown in Figure 1b. Figure 1c shows the channel region of another device (*Device-B*) having uniform phosphorus (*P*) doping (*N*_D_ ≈ 1 × 10^18^ cm^−3^) in the channel region, along with the source and drain leads. Similar doping condition is valid for the source and drain leads for *Device-A*. The potential wells induced by several ionized *P*−donors in the channel are schematically illustrated in Figure 1d. Considering the channel dimensions and dopant concentration, it is estimated that around 5 *P*−donors are present in the channel (labeled as *P*i, with i = 1–5).

### 2.2. Device Configuration for Theoretical Calculations

**Figure 2:**
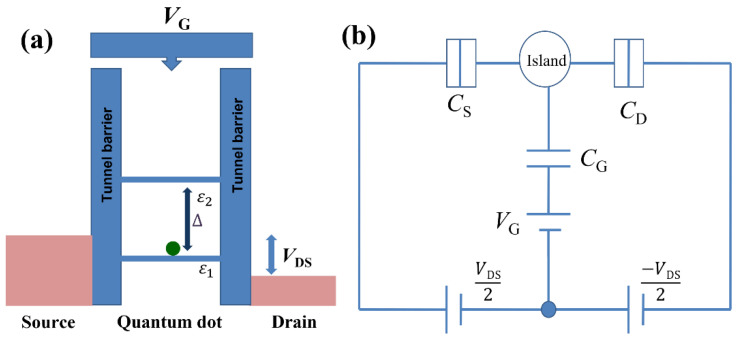
(**a**) Schematic potential diagram of a double-barrier QD system with the tunnel barriers also controlled by the gate voltage. (**b**) Equivalent electrical circuit model of a QD in the double-barrier configuration with symmetric bias.

The primary interest of this report is to solve the tunneling current through a semiconductor SET operating in the quantum regime, in a realistic device configuration, as explained in the previous section. A schematic potential profile of a QD with discrete energy levels, weakly coupled to the two electron reservoirs via tunnel barriers, in thermal equilibrium, is shown in Figure 2a. The discreteness of the energy levels in the QD is considered either in the frame of the quantum-size effect for a situation expected in *Device-A* or in the frame of the discrete energy spectrum of *P*−donors for cases such as expected in *Device-B*. The equivalent electrical circuit under consideration is shown schematically in Figure 2b, together with the applied bias voltage *V*_DS_ and the gate voltage *V*_G_. Both reservoirs and gate electrode are capacitively connected to the QD through capacitances *C*_S_, *C*_D_*,* and *C*_G_, respectively, with the total capacitance of the system being: *C*_∑_ *= C*_S_ *+ C*_D_ *+ C*_G_. In this device, the transfer of electrons from reservoirs to the QD or vice versa is governed mainly by the potential differences between the leads and the QD. We chose the reference electrostatic potential in such a way that the energy levels in the QD are independent of the bias voltages [32]. On the contrary, the Fermi energies of the leads are described as a function of the different capacitances and applied voltages as:

(1a) $$E_{S}^{F}=e\frac{2C_{D}+C_{G}}{2C_{\Sigma }}V_{\mathrm{DS}}-e\frac{C_{G}}{C_{\Sigma }}V_{G}$$

(1b) $$E_{D}^{F}=-e\frac{2C_{S}+C_{G}}{2C_{\Sigma }}V_{\mathrm{DS}}-e\frac{C_{G}}{C_{\Sigma }}V_{G}$$

We define the problem under the conditions that ensure the observation of quantum transport features: (a) thermal energy (*k*_B_*T*) is much smaller than the level spacing (*Δ*) of the QD, while this level spacing itself must be smaller than the charging energy (*E*_C_ *= e*^2^*/2C*_∑_) of the QD; (b) tunnel resistance (R_t_) of both barriers is greater than quantum resistance (*h/e*^2^ *=* 25.81 kΩ) which ensures suppression of the higher-order tunneling processes; (c) a continuum of states in both electron reservoirs is assumed, ensuring the absence of discreteness in the local density of states (LDOS) of the leads [1]. Moreover, all types of internal relaxations and electron-electron interactions within the QD are also neglected in this model.

### 2.3. Theoretical Formalism

The description of the tunneling transport through a QD is performed using the Anderson Hamiltonian of a QD having single-particle energy levels connected to electron reservoirs (source and drain) [31,32,40]:

(2) $$H=H_{\mathrm{Dot}}+H_{S\left(D\right)}+H_{T}$$

where the Hamiltonian for coupling of the QD to source and drain reservoirs is *H*_T_, while the Hamiltonians for an ideal, isolated QD and for the source (drain) reservoirs are *H*_Dot_ and *H*_S(D)_, respectively. Here:

(3a) $$H_{S\left(D\right)}=\underset{k\sigma ,i\in S\left(D\right)}{\sum }\varepsilon_{\mathrm{ki}}c_{k\sigma i}^{†}c_{k\sigma i}$$

(3b) $$H_{\mathrm{Dot}}=\underset{l\sigma \;}{\sum }\varepsilon_{l\sigma j}b_{l\sigma j}^{†}b_{l\sigma j}+E_{C}N_{e}{}^{2}+N_{e}U_{\mathrm{ext}}$$

(3c) $$H_{T}=\underset{i\in S\left(D\right)}{\sum }\underset{\mathrm{kl}\sigma }{\sum }T_{\mathrm{kl}\sigma ,i}c_{k\sigma i}^{†}b_{l\sigma j}+\left(h.c\right)$$

(3d) $$H_{T}=\underset{i\in S\left(D\right)}{\sum }\underset{k\sigma ,\psi \phi }{\sum }T_{k\sigma ,\psi \phi ,i}c_{k\sigma i}^{†}|\psi \rangle \langle \phi |+\left(h.c\right)$$

(3e) $$T_{k\sigma .\psi \phi }=\underset{l}{\sum }T_{\mathrm{kl}\sigma ,i}\langle \psi \left|b_{l\sigma j}\right|\phi \rangle$$

Here $\varepsilon_{\mathrm{ij}}\;\left(eV\right)$ are the single-particle states of the QD, whereas $|\psi \rangle$ is the many-body eigenstate of the QD, differing from $|\phi \rangle$ by a single extra electron on the jth level. In addition,$\;U_{\mathrm{ext}}=\underset{r\in S,D,G}{\sum }C_{r}V_{r}/C_{\Sigma }\;$defines the electrostatic work performed to add extra electrons *N*_e_ into the QD.

Following the Fermi golden rule for the total transition rate governed by transition matrix T between the QD’s energy level and the reservoir:

(4) $$\Gamma =\frac{2\pi }{\hbar }\sum |\langle \psi_{\mathrm{final}}\left|T\right|\psi_{\mathrm{initial}}\rangle {|}^{2}\delta \left(\epsilon_{\mathrm{initial}}-\epsilon_{\mathrm{final}}\right)$$

We obtain the tunneling rate from QD to reservoir or vice-versa as [31]:

(5a) $$\gamma_{\phi \to \psi ,\;i}=\frac{2\pi }{\hbar }\underset{k\sigma }{\sum }f\left(\epsilon_{\mathrm{ki}}\right){\left|T_{k\sigma ,\phi \psi ,i}^{*}\right|}^{2}\delta \left(\epsilon_{\psi }-\epsilon_{\phi }-\varepsilon_{k,i}-eV_{i}\right)$$

(5b) $$\gamma_{\psi \to \phi ,\;i}=\frac{2\pi }{\hbar }\underset{k\sigma }{\sum }\left(1-f\left(\epsilon_{\mathrm{ki}}\right)\right){\left|T_{k\sigma ,\psi \phi ,i}\right|}^{2}\delta \left(\epsilon_{\psi }-\epsilon_{\phi }+\varepsilon_{k,i}+eV_{i}\right)$$

Considering a symmetric-bias configuration, $V_{i\left(S,D\right)}=\frac{V_{\mathrm{DS}}}{2}\left(\frac{-V_{\mathrm{DS}}}{2}\right)$, the total tunneling rates can be finally written as [32]:

(6a) $$W_{\phi \to \psi ,j}=\Gamma_{j}^{S}f\left({\tilde{\varepsilon }}_{j}–E_{S}^{F}\right)\left(2-n_{j}\right)+\Gamma_{j}^{D}f\left({\tilde{\varepsilon }}_{j}–E_{D}^{F}\right)\left(2-n_{j}\right)$$

(6b) $$W_{\psi \to \phi ,j}=\Gamma_{j}^{S}\left[1-f\left({\tilde{\varepsilon }}_{j}–E_{S}^{F}\right)\right]\left(n_{j}\right)+\Gamma_{j}^{D}\left[1-f\left({\tilde{\varepsilon }}_{j}–E_{D}^{F}\right)\right]\left(n_{j}\right)$$

Here, *W* is the total tunnel rate via the jth single-particle energy level for adding an extra electron to configuration $|\phi \rangle$ at the jth level, with occupation number (*n*_j_ = either ‘0′ or ‘1′) and energy redefined as ${\tilde{\varepsilon }}_{j}=\varepsilon_{j}+E_{C}\;\left(\mathrm{eV}\right)$, connected to both the reservoirs. Here, $\Gamma_{j}^{S\;\left(D\right)}$ is the bare tunneling rate of the respective energy level coupled with source (drain), whereas ‘$f\left(x\right)$’ defines the Fermi function at the temperature *T*:

$$f\left(x\right)=\frac{1}{\left(1+e^{\frac{x}{k_{B}T}}\right)}$$

All the tunnel rates W,Γ, and γ bear the same unit, s^−1^. 

The generalized total transition rates, as described in Equation (6a,b), are utilized and analyzed for two possible situations:

(i) For infinitely high tunnel barriers, it would suffice to consider $\Gamma_{j}^{S\;\left(D\right)}$= $\Gamma^{S\left(D\right)}$ = constant.
(ii) For finite and bias-dependent barrier, the bare tunneling rate is varying with the bias voltage as presented below:

(7) $$\Gamma_{j}^{S\left(D\right)}=\frac{1}{R_{t\left(j\right)}e^{2}}\frac{\varepsilon_{j}\;–\;E_{S\left(D\right)}^{F}}{1-\exp\left(\beta (\varepsilon_{j}\;–\;E_{S\left(D\right)\;}^{F})\right)}$$

with $R_{t\left(j\right)}$ (Ω) being the barrier resistance dependent on the energy levels.

Following the rate equation method, the occupation probability (*P*) can be represented as [41]:

(8) $$\frac{\;dP_{\phi }}{dt}=\underset{\psi }{\sum }W_{\psi \to \phi }P_{\psi }-W_{\phi \to \psi }P_{\phi }$$

The steady state occupation probabilities *P* can be found by iterating Equation (8) with the normalization conditions, $\underset{\phi }{\sum }P_{\phi }=1\mathrm{and}\;dP/\mathrm{dt}=W.P=0.$

To solve for the occupation probabilities of each configuration$|\varphi \rangle$ for a particular bias and gate voltage, the current through either of the barriers can be calculated using Equations (6)–(8). Steady-state current through both the junctions should be equal for symmetric-barrier configurations and is given by:

(9) $$I_{S}=\left|e\right|\underset{\phi }{\sum }\underset{\psi }{\sum }W_{\phi \to \psi }^{S}P_{\phi }$$

In case of *N* accessible energy levels in the QD within the bias window of the SET device, Equation (8) can be generalized as:

(10) $$\left[\begin{array}{cccccc} -\sum_{j=1}^{N}W_{0\to 1,j} & W_{1\to 0,1} & W_{1\to 0,2} & W_{1\to 0,3} & . . . & W_{1\to 0,N}\\ W_{0\to 1,1} & -W_{1\to 0,1} & 0 & 0 & . . . & 0\\ W_{0\to 1,2} & 0 & -W_{1\to 0,2} & 0 & . . . & 0\\ W_{0\to 1,3} & 0 & 0 & -W_{1\to 0,3} & . . . & 0\\ . & . & . & . & . . . & .\\ . & . & . & . & . . . & .\\ . & . & . & . & . . . & .\\ W_{0\to 1,N} & 0 & 0 & 0 & . . . & -W_{1\to 0,N} \end{array}.\right]\left[\begin{array}{c} P_{0}\\ P_{1}\\ P_{2}\\ P_{3}\\ .\\ .\\ .\\ P_{N} \end{array}\right]=0$$

where $P_{j}=\frac{W_{01\_j}\prod_{\begin{array}{c} m=1\\ m\neq j \end{array}}^{N}W_{10\_j}}{W_{\mathrm{sum}}}$ are the corresponding occupation probabilities of the jth electronic level with

$$W_{\mathrm{sum}}=\overset{N}{\underset{j=1}{\sum }}\overset{N}{\underset{m\;\neq j}{\prod }}W_{\phi \to \psi ,\;m}W_{\psi \to \phi ,\;j}+\overset{N}{\underset{j=1}{\prod }}W_{\phi \to \psi ,\;j}$$

In order to understand the underlying physics behind the above formalism better and to visualize the scenarios described by these equations, we discuss some simple implementations of the approach in Section 3.2, starting with a few experimental results that can be explained by such a formalism in Section 3.1.

## 3. Results and Discussion

### 3.1. Experimental Evidences for the Effect of Variable Tunnel Barriers of a QD

**Figure 3:**
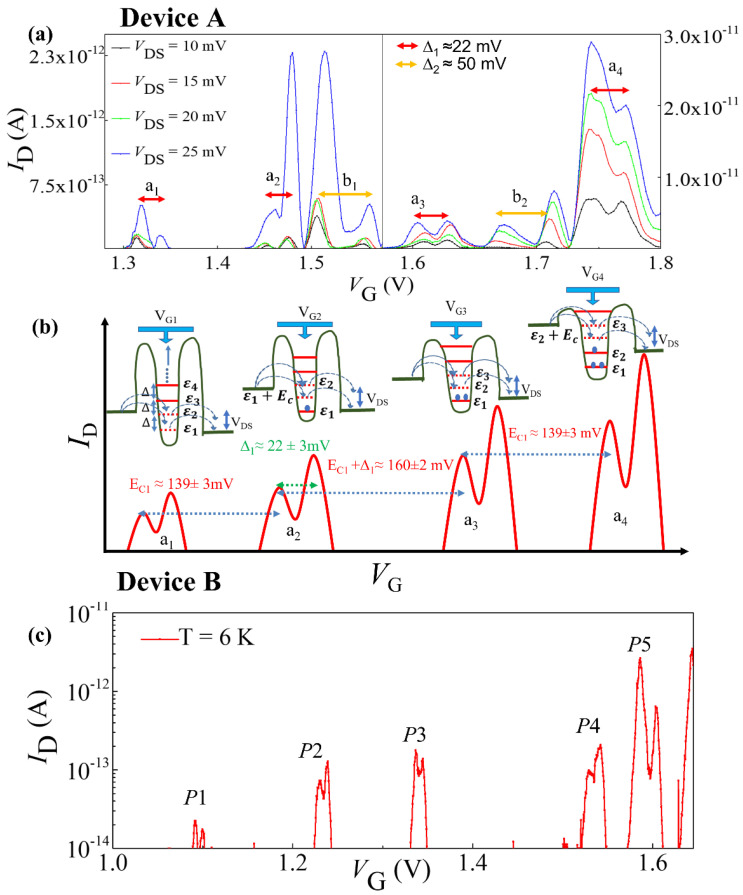
Device A (**a**) *I*_D_−*V*_G_ characteristics measured at T = 5 K, depicting also the single-electron-tunneling current peaks *a*_1_–*a*_4_ and *b*_1_, *b*_2_, likely originated from QD_1_ and QD_2_, respectively. (**b**) Schematic summarization of tunneling transport through the QD_1_, suggesting the variable-barrier QCB. Device B (**c**) *I*_D_−*V*_G_ plot shows the SET transport through isolated *P*−donors in uniformly-doped SOI-FET channels.

The structures and technical details for the devices experimentally studied in this work, specifically, SOI-FETs with undoped channel (*Device-A*) or uniformly-doped channel (*Device-B*) are explained in Figure 1. As mentioned earlier, in *Device A*, although having the channel nominally undoped, QDs may be induced by some degree of roughness in the channel region. This is depicted as QD_1_ and QD_2_ in Figure 1a and as broader potential wells in Figure 1b. *I*_D_−*V*_G_ characteristics measured at low-temperature (5 K) for *Device-A* are presented in Figure 3a. In this figure, SET current peaks can be identified, labeled as *a*_1_, *a*_2_, *a*_3_, *a*_4_, *b*_1_, and *b*_2_. Each of these current peaks has several associated sub-peaks. For *a*_1_–*a*_4_ peaks, the gap between consecutive subpeaks (*Δ*_1_) is ~22 ± 3 mV, while for *b*_1_, *b*_2_ peaks, the same gap (*Δ*_2_) is ~50 ± 2 mV. These associated subpeaks are most likely due to transport mediated by the excited states of their respective QD [42]. Hence, it is reasonable to assume that the *a*_1_–*a*_4_ peaks are associated with QD_1,_ while the *b*_1_ and *b*_2_ peaks are associated with QD_2_. The gap between *a*_1_ and *a*_2_ (*E*_C1_) is 139 ± 3 mV. Similar gap is observed between *a*_3_ and *a*_4_. The energy gap between *a*_2_ and *a*_3_ is 160 ± 2 mV, which is also the sum of *E*_C1_ and *Δ*_1_. This is the clear indication of quantum Coulomb blockade phenomenon.

In addition, we observed that the current intensity is gradually increased while we move from *a*_1_ toward *a*_4_ peaks, which strongly suggests gradual increment of the tunnel rate with increasing gate voltage. The relation between *a*_1_–*a*_4_ peaks is schematically presented in Figure 3b in correlation with a simplified representation of transport and electrical characteristics. This simplified picture is emphasizing the expected behavior under the observation of variable-barrier quantum Coulomb blockade in the QD_1_ system.

The *I*_D_−*V*_G_ characteristic of *Device-B* measured at *T* = 6 K is presented in Figure 3c. The device configuration of *Device-B* is basically uniformly doped MOSFET in SOI configuration as presented in Figure 1d. Five single-electron-current peaks are observed with irregular spacing before the onset of FET current. These current peaks are separated by Coulomb energy. The spacing between these current peaks are irregular, which generally originated from different quantum dots. Considering the devices configuration of the Device-B, these quantum dots are most likely due to donor present (*P*−donor in this case) in the channel region of the device as reported earlier [37,38,39,43]. Due to the different positions of the donor atoms in the channel region, all donors have different barrier parameters and that can be controlled by the gate voltage. The origin of five SET peaks can be directly correlated to the existence of 5 *P*−donors in the channel region of the device as estimated from the device designing. The schematic dopant distribution and potential configuration of this device structure are shown in Figure 1c,d. We also observed transport through the excited state of the donor QD with the average separation of excited state from the ground state of the donor as 8 ± 2 mV. This separation is tentatively consisted with the energy spectrum of the *P*−donor [44]. In addition, we also observed that the heights of the current peaks are gradually enhanced with the increasing gate voltage. This suggests that the tunneling rates are also tuned by the gate voltage even in the case of donor-induced QDs.

### 3.2. Numerical Analysis of Electron Transport

In this section, we numerically demonstrate the transport properties of the single-QD based SET device in the QCB regime, under the condition that multiple energy levels are accessible during the electron transport. After calculation of the generalized transition matrix and occupation probability, the parameters described earlier will be used for the study of two-level and three-level cases. For the numerical calculation, we consider both infinite- (i.e., constant-rate) and variable-barrier conditions for the single electron tunneling processes.

**Figure 4:**
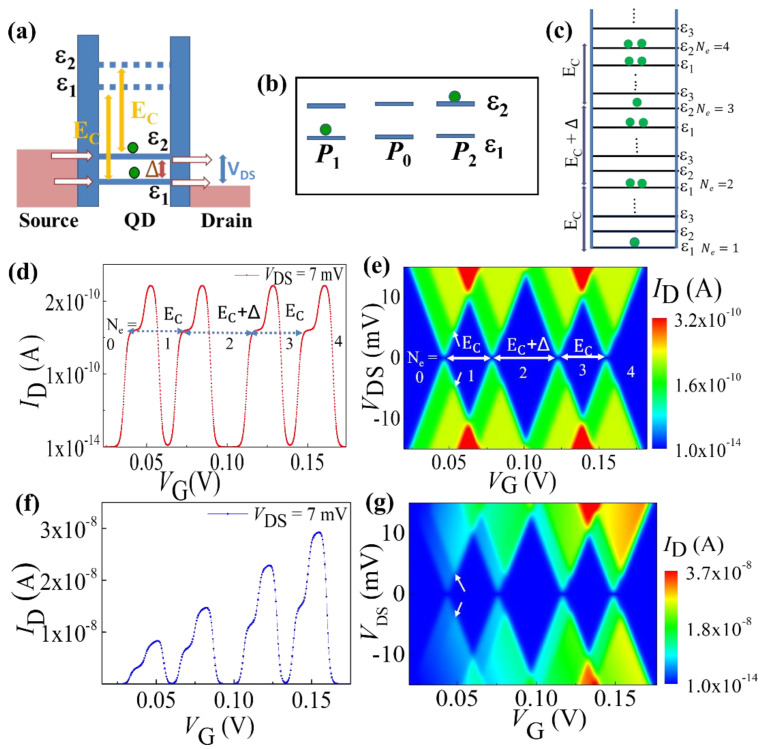
(**a**) Schematic representation of a QD with two energy levels accessible in the bias window, connected with source and drain reservoirs. (**b**) Probable transitions from $|\phi \rangle$ (*P*_0_) to $|\psi \rangle$(*P*_1_ and *P*_2_) are shown. (**c**) Schematic representations of the successive incorporation of electrons in the QD for the quantum Coulomb blockade case. (**d**,**e**) *I*_D_−*V*_G_ characteristics and stability diagram of a SET system with constant bare tunnel rates, respectively. (**f**,**g**) *I*_D_−*V*_G_ characteristics and stability diagram of a practical SET setup with biasing-dependent tunnel rates, respectively. Arrows indicate the onset of transport through a new energy level.

**i.**  Electron Transport through Two Energy Levels:

The tunneling matrix and probabilities are calculated for the QD coupled to the source and drain reservoirs, when two energy levels are accessible within the bias window, as shown in Figure 4a. The two energy levels are marked as $\varepsilon_{1}$ and $\varepsilon_{2}$, corresponding to j = 1 and j = 2, respectively. The tunneling rates corresponding to the tunneling transition in the 1st charge states are:

(11) $$W_{0\to 1,1}=\Gamma_{1}^{S}\;2f\left({\tilde{\varepsilon }}_{1}\;–\;E_{S}^{F}\right)+\Gamma_{1}^{D}\;2f\left({\tilde{\varepsilon }}_{1}\;–\;E_{D}^{F}\right)$$

(12) $$W_{0\to 1,2}=\Gamma_{2}^{S}\;2f\left({\tilde{\varepsilon }}_{2}\;–\;E_{S}^{F}\right)+\Gamma_{2}^{D}\;2f\left({\tilde{\varepsilon }}_{2}\;–\;E_{D}^{F}\right)$$

(13) $$W_{1\to 0,1}=\Gamma_{1}^{S}\;\left[1-f\left({\tilde{\varepsilon }}_{1}\;–\;E_{S}^{F}\right)\right]+\Gamma_{1}^{D}\left[1-f\left({\tilde{\varepsilon }}_{1}\;–\;E_{D}^{F}\right)\right]$$

(14) $$W_{1\to 0,2}=\Gamma_{2}^{S}\;\left[1-f\left({\tilde{\varepsilon }}_{2}\;–\;E_{S}^{F}\right)\right]+\Gamma_{2}^{D}\left[1-f\left({\tilde{\varepsilon }}_{2}\;–\;E_{D}^{F}\right)\right]$$

After replacing the tunnel rates defined in Equations (11)–(14), the Equation (8) can be written for the steady-state condition as:

(15) $$\left[\begin{array}{ccc} -\left(W_{0\to 1,\;1}+W_{0\to 1,\;2}\right) & W_{1\to 0,\;1} & W_{1\to 0,2}\\ W_{0\to 1,\;1} & -\left(W_{1\to 0,\;1}\right) & 0\\ W_{0\to 1,\;2} & 0 & -\left(W_{1\to 0,2}\right) \end{array}\right]\left[\begin{array}{c} P_{0}\\ P_{1}\\ P_{2} \end{array}\right]=\left[\begin{array}{c} 0\\ 0\\ 0 \end{array}\right],$$

where *P*_0_, *P*_1_, and *P*_2_ are the occupation probabilities of respective electronic configurations (0,0), (1,0), and (0,1) as schematically depicted in Figure 4b. These are expressed as:

(16) $$P_{0}=\left(W_{1\to 0,\;1}W_{1\to 0,\;2}\right)/W_{\mathrm{sum}}$$

(17) $$P_{1}=\left(W_{0\to 1,\;1}W_{1\to 0,\;2}\right)/W_{\mathrm{sum}}$$

(18) $$P_{2}=\left(W_{1\to 0,\;1}W_{0\to 1,\;2}\right)/W_{\mathrm{sum}}$$

(19) $$W_{\mathrm{sum}}=W_{1\to 0,\;1}W_{1\to 0,\;2}+W_{0\to 1,\;1}W_{1\to 0,\;2}+W_{1\to 0,\;1}W_{0\to 1,\;2}\;$$

Now, the total tunneling current for this configuration can be described as:

(20) $$I=-(W_{0\to 1,1}+W_{0\to 1,2})P_{0}+\left(W_{1\to 0,1}\right)P_{1}+(W_{1\to 0,\;2})P_{2}$$

The systematic electron incorporation in this device configuration is schematically presented in Figure 4c following the quantum Coulomb blockade phenomenon. This process is considered in the numerical model. We consider a QD with a constant energy separation between successive states of *Δ* = 3 meV and *E_C_* = 10.66 meV for numerical calculation of the characteristics of the device. All these calculations are performed at low temperature of *T* = 4 K, comparable to the condition for the experimental data. Here, we accounted for both situations: (i) the tunneling rate is constant considering the infinite barrier height and (ii) tunneling rate is varying with the applied gate voltage. For the first case, the calculated *I*_D_-*V*_G_ characteristic for different charge states is plotted in Figure 4d. Two separate current sub-peaks within a peak can be assigned to SET transport involving the ground state and the 1st excited state. The level separation of the sub-peaks, *Δ*, and an alternative energy separation of *E*_C_ and *E*_C_ + *Δ* are clear signatures of quantum Coulomb blockade. The stability diagram (i.e., the plot of *I*_D_ in the *V*_G_−*V*_DS_ plane) is shown in Figure 4e, where the excited-state features are also clearly observed as marked by white arrows in the first charge state. Successive incorporation of charges in the device is visible in the stability diagram as *N*_e_ changes from 0 to 4.

Now, we discuss about the more realistic situation where the tunnel barriers modulate according to the biasing condition. The simulated results with the modified formula for variable tunnel rate as presented in Equation (7) are shown in Figure 4f,g. The impact of systematic increment of the gate voltage on the tunnel barriers can easily be noticed from Figure 4f as the systematic increment of the SET current peak heights. This supports the interpretation of the observed SET current features in our experimental data. The stability diagram of the device is also simulated and presented as a contour plot of |*I*_D_| in *V*_G_−*V*_DS_ plane (Figure 4g). The systematic enhancement of the current intensity of conducting region of the stability diagram is clearly visible when *N*_e_ changes from 0 to 4, consistent with the recent experimental observations.

**Figure 5:**
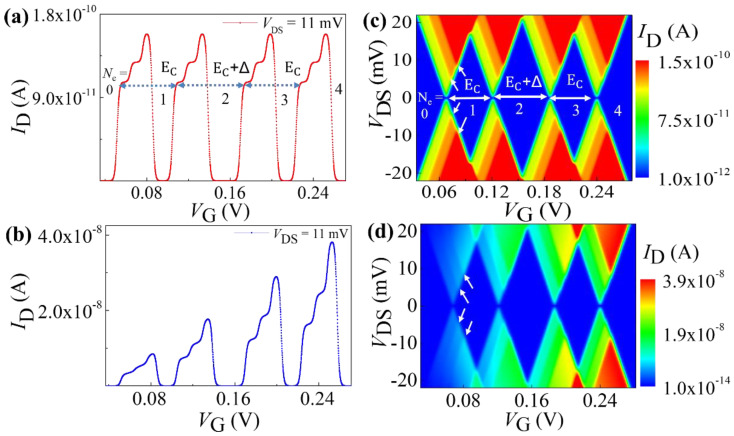
(**a**,**b**) Simulated *I*_D_−*V*_G_ characteristics with three energy levels within the bias window for infinite- and variable-height barrier devices, respectively. (**c**,**d**) Simulated stability diagram for the same device configurations. Arrows indicate the onset of transport through a new energy level.

**ii.**  Electron Transport through Three Energy Levels:

In this section, we extend the model for three spin-degenerate states available for charge transport through the QD. The tunneling probabilities and current are calculated using the matrix mentioned in the above sections. The simulated *I*_D_*-V*_G_ features for constant- and variable-height tunnel barriers are presented in Figure 5a,b, respectively. In each SET current peak, we observed three subpeaks as expected due to the accessibility of three energy levels in the bias window. The realistic device feature for the variable-height barrier case is also clearly observed in the Figure 5b. The stability diagram corresponding to infinite- and variable-height barrier cases are presented in Figure 5c,d, respectively. The systematic incorporation of additional energy levels in the transport path is depicted by white arrows in Figure 5c,d. The features of quantum Coulomb blockade and the differences between infinite- and variable-height barrier configurations are clearly visible in these figures, confirming the feasibility of our approach towards the qualitative replication of the experimental data.

## 4. Conclusions

We presented low-temperature transport characteristics of nanoscale single-electron tunneling transistors made in SOI-FET configuration using silicon quantum dots and *P*−donor induced QDs. These devices depicted the quantum Coulomb blockade effect along with the influence of bias dependent tunnel barriers. To support the experimental findings, a generalized theoretical formalism for variable barrier quantum Coulomb blockade phenomenon is developed by modifying the rate equation approach. We emphasized on the quantum transport regime, showing how the addition energy for successive electron transfer events oscillates between *E*_C_ and *E*_C_ + *Δ* correlated to spin degeneracy of the energy levels along with the modification of tunnel rate due to variation in the tunnel barrier. To qualitatively reproduce the experimental findings of realistic devices, we have numerically calculated the current voltage characteristics for the constant and variable tunnel barrier conditions. We showed that the numerical results for QD with two and three levels accessible for tunneling transport. The modified theoretical formalism closely replicates the nano-scaled SET devices fabricated in two-dimensional electron gas (2DEG) systems, semiconductor QDs, and dopants as QDs.

For the practical operation towards target functionalities, it becomes important to account for the effect of barrier height in the design of the device geometry. The approach described here can help to understand such nanoscale devices in a more appropriate manner, allowing the development of useful functionalities towards low-power electronics, single-electron memories, or single-charge advanced sensing devices.

## Data Availability

The data presented in this study are available upon request from the corresponding author.
